# Signal Activity Detection for Fiber Optic Distributed Acoustic Sensing with Adaptive-Calculated Threshold

**DOI:** 10.3390/s22041670

**Published:** 2022-02-21

**Authors:** Lilong Ma, Tuanwei Xu, Kai Cao, Yinghao Jiang, Dimin Deng, Fang Li

**Affiliations:** 1 State Key Laboratory of Transducer Technology, Institute of Semiconductors, Chinese Academy of Sciences, Beijing 100083, China; malilong@semi.ac.cn (L.M.); caokai@semi.ac.cn (K.C.); bjhdjyh@semi.ac.cn (Y.J.); ddm911@semi.ac.cn (D.D.); lifang@semi.ac.cn (F.L.); 2 College of Materials Science and Opto-Electronic Technology, University of Chinese Academy of Sciences, Beijing 100049, China

**Keywords:** distributed acoustic sensing, signal activity detection, adaptive-calculated threshold, short term energy analysis

## Abstract

The key point on analyzing the data stream measured by fiber optic distributed acoustic sensing (DAS) is signal activity detection separating measured signals from environmental noise. The inability to calculate the threshold for signal activity detection accurately and efficiently without affecting the measured signals is a bottleneck problem for current methods. In this article, a novel signal activity detection method with the adaptive-calculated threshold is proposed to solve the problem. With the analysis of the time-varying random noise’s statistical commonality and the short-term energy (STE) of real-time data stream, the top range of the total STE distribution of the noise is found accurately for real-time data stream’s ascending STE, thus the adaptive dividing level of signals and noise is obtained as the threshold. Experiments are implemented with simulated database and urban field database with complex noise. The average detection accuracies of the two databases are 97.34% and 90.94% only consuming 0.0057 s for a data stream of 10 s, which demonstrates the proposed method is accurate and high efficiency for signal activity detection.

## 1. Introduction

The advanced perception technology is the source of big data, the foundation of artificial intelligence development, and the key technical support to construct a smart earth, a smart ocean and a smart city [1]. The sensing capabilities of high sensitivity, long distance and high space-time accuracy enable DAS as a hot point in sensing technology, which has been widely applied in long-distance perimeter security [2,3,4], oil or gas pipeline invasion [5,6,7], seismic detection [8,9,10], natural resource exploration [11,12] and other applications [13,14,15]. The pivotal technology for the analysis of the DAS data stream is to separate the signals and environmental noise accurately and efficiently. It should be noted that the sampling rate of the data acquisition card used in the DAS system is usually hundreds of megahertz. Hence, it is urgent to develop a low computational complexity, fast response, high accuracy and strong robustness signal activity detection for real-time signal analysis.

With the runtime of several milliseconds, time domain characteristics of zero-crossing rate (ZCR) and short-term energy (STE) are the most common indicators for signal activity detection. K. Liu et al. applied the dual-crossing method to part signals and environmental noise depending on ZCR [16,17]. Due to the principle of counting the number of zero-crossing values in several fixed time blocks of the data stream, it is more sensitive to high-frequency signals and loses low-frequency signals. A real-time signal detection based on an STE crossing level algorithm with an average accuracy of 84.4% was implemented in [18], and a dual-threshold method combined by STE and ZCR with an average accuracy of 76.45% was presented in [19]. Both predefined thresholds are set by the environmental noise at the initial moment, which leads to inaccurate detection for the varying noise.

The developed signal activity detection methods relying on frequency domain characteristics can better highlight differences between signals and environmental noise. Various frequency features emerged such as long-term signal variability (LTSV) feature, long-term spectral flatness measure (LSFM) feature and long-term spectral variability measure (LSVM), and the average detection accuracy based on the three features could reach more than 85% with the runtime about 1 second [19,20,21,22]. However, the time resolution is limited to the long window, inducing non-negligible deviations from the actual onset and endpoint of the signal. Letter [23] put forward a novel detection algorithm based on a high-pass convolution window finite impulse response filter to remove environmental noise, and experimental results showed that the proposed detection outperformed the wavelet-based method. Further, a low-order high-pass filter whose cut-off frequency could rapidly adapt to environmental noise was designed [24,25], but low-frequency components of signal were also filtered out inevitably.

With a series of features emerging in time and frequency domains, researchers attempted to derive a more suitable detection method by integrating both of these domain features. As described in [26], since the amplitude of the high-frequency components had a noticeable jump when the signal occurred in a short time, a short-time Fourier transform (STFT)-based method was applied to detect the signal with the minimum runtime of 0.145 s. However, only two signals of climbing the fence and knocking the cable were verified. The signal was decomposed into intrinsic mode functions (IMFs) to realize detection using the threshold judged by the first five frames as noisy fragments of the data stream and the accuracy was 87.29% when the signal-to-noise ratio (SNR) was 0 dB [27]. Due to the zooming property, the wavelet method provides high accuracy for signal analysis, and paper [28] focused on wavelet transform (WT) to realize signal activity detection and an average accuracy of 90.1% was obtained with the update threshold of noise at the first ten frames of the data stream. However as illustrated in [23], this method with the runtime in several seconds was inefficient for the high computation complexity of wavelet decomposition in multiple layers.

In general, current methods mainly rely on the predefined threshold set only by the environmental noise at the beginning of the data stream, which ignore the time-varying nature of the environmental noise, and there are some methods of adaptive thresholds, but the accuracies of the thresholds need to be improved. Facing the application requirements of DAS for accurate and efficient signal activity detection, a novel method with the analysis of the time-varying random noise’s statistical commonality and the real-time data stream’s STE is proposed to solve the above problems. Firstly, the principle of simulation data is illustrated, and the signal activity detection algorithm with the adaptive-calculated threshold is explained in detail. Secondly, the detection results of the simulation database with the SNR of 5 to 25 dB are compared with the LSFM and the STFT methods, and the proposed method has the best average detection accuracy of 97.34%. Finally, field experiments with complex urban noise are implemented, and the two reference methods have poor performances, and a detection accuracy of 90.94% only consuming 0.0057 s for a data stream of 10 s is obtained by the proposed method. The results demonstrate that the proposed methods meet the application requirements of DAS for signal activity detection.

The paper is arranged as the follows. Section 2 is the principal part, which includes the principle of the simulation data stream measured by DAS and the signal activity detection algorithm with the adaptive-calculated threshold. Section 3 verifies the effectiveness of the proposed method with the simulated database and the actual database compared with the LSFM and the STFT methods. Section 4 comes to the discussion. Finally, Section 5 is the conclusion.

## 2. Materials and Methods

This section consists of two parts. Firstly, to study signal activity detection with the adaptive-calculated threshold at different SNR levels, the man-made vibrations of ground measured by DAS are simulated by the single degree of free system and simulated data streams with SNR of 5 to 25 dB are obtained. Secondly, the signal activity algorithm detection with the adaptive-calculate threshold is presented in detail.

### 2.1. Simulated Data Stream

**Figure A1:**
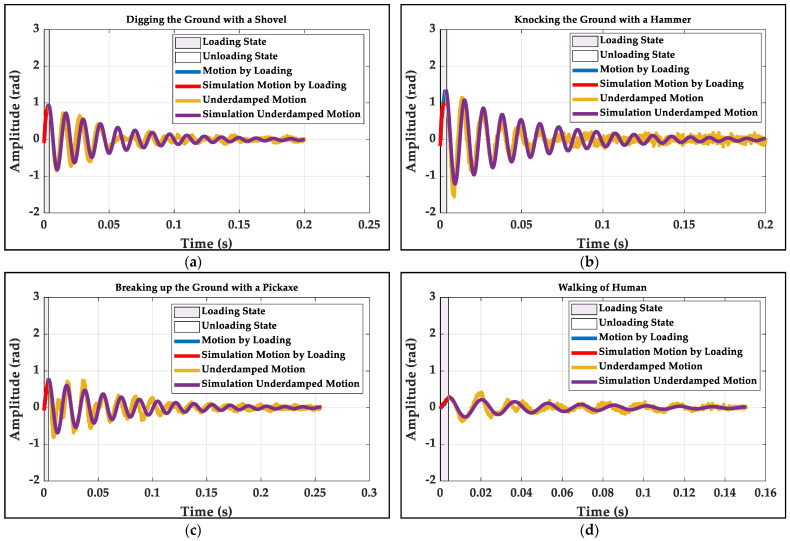
The actual and simulated vibrations of the four kinds of signals: (**a**) digging the ground with a shovel; (**b**) knocking the ground with a hammer; (**c**) breaking up the ground with a pickaxe; (**d**) walking of human.

A simple method summarizing the ground vibrations measured by DAS is presented referring to the single degree of free system (the motion equation) of the drill in the percussive system propelled into rocky ground [29]. Based on three aspects, firstly, the most ground vibrations are caused by sudden loading; secondly, the ground is not elastic, and the soil damping coefficient is less than 1 [30,31,32,33]; thirdly, DAS measures vibration signals in a single direction. The vibration has two-stage displacements when the source of the vibration acts on the ground. The displacement by incident loading and the underdamped motion during unloading are expressed as:

(1) $$u(t)=\left\{ \begin{array}{l} 1-e^{-2(\beta /\tau )t}\times [\frac{1}{\sqrt{\alpha -1}}\sin(\frac{2\sqrt{\alpha -1}}{\alpha }\frac{\beta }{\tau }t)+\cos(\frac{2\sqrt{\alpha -1}}{\alpha }\frac{\beta }{\tau }t)](\mathrm{loading})\\ e^{-(2/\alpha )(\beta /\tau )t}\times [(\frac{\alpha }{2\sqrt{\alpha -1}}\frac{\tau }{\beta })\overset{•}{u_{0}}\times \sin(\frac{2\sqrt{\alpha -1}}{\alpha })\frac{\beta }{\tau }t+\frac{1}{\sqrt{\alpha -1}}u_{0}\times \sin(\frac{2\sqrt{\alpha -1}}{\alpha }\frac{\beta }{\tau }t)+u_{0}\times \cos(\frac{2\sqrt{\alpha -1}}{\alpha }\frac{\beta }{\tau }t)](\mathrm{unloading}) \end{array}\right.$$

where α equals $4\;km_{b}/{\left(\rho cA\right)}^{2}$ and β equals kτ/ρcA, and $m_{b}$, ρ, c, A, k, τ, $u_{0}$ and $u^{\cdot }{}_{0}$ are the soil mass of loading by incident loading, the density of soil, the wave velocity propagating in the ground, the cross-section area between ground and vibration source, the elastic stiffness coefficient of soil, the duration of the sudden loading, the maximum displacement of ground vibration and the first derivative of maximum displacement, respectively. The rationality of the simulation data is explained in Appendix A and four kinds of ground vibrations collected in this article are simulated (digging the ground with a shovel, knocking the ground with a hammer, breaking up the ground with a pickaxe and walking of a human) shown in the Figure A1 in Appendix A.

In the actual data collection work, the environmental noise is inevitably measured by DAS, and the real-time data stream can be expressed as:

(2) $$x(t)=\left\{ \begin{array}{ll} u(t)+v(t) & u(t)\neq 0\\ v(t) & u(t)=0\; \end{array}\right.$$

where x(t), u(t) and v(t) are the data stream, ground vibration and the additive noise (the simulated noise includes white noise, pink noise and Brownian noise to mimic random noise measured by DAS dominated by low-frequency components).

### 2.2. Algorithm of Signal Activity Detection with the Adaptive-Calculated Threshold

**Figure 1:**
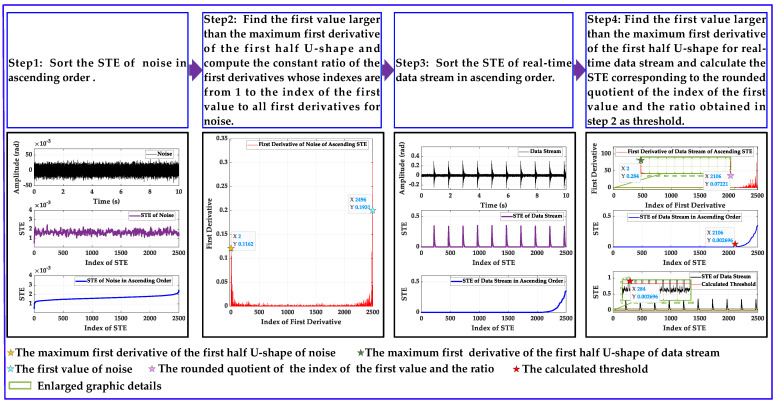
The flow chart of signal activity detection with the adaptive-calculated threshold and the calculation results of every step.

Figure 1 illustrates the flow chart of the proposed signal activity detection with the adaptive-calculated threshold and the calculation results of every step. This algorithm mainly consists of the following steps:

Step 1: Analyze the STE of a certain amount of noise. Firstly, calculate the STE of noise ($E_{noise}=[E_{1},E_{2},E_{3},\ldots ,E_{n}]$). Secondly, sort the STE in ascending order ($E_{noise}^{\prime }=[E_{1}{}^{\prime },E_{2}{}^{\prime },E_{3}{}^{\prime },\ldots ,E_{m}{}^{\prime }]$). Finally, calculate the first derivatives of the sort ($\overset{\cdot }{E_{noise}^{\prime }}=[\overset{\cdot }{E_{1}{}^{\prime }},\overset{\cdot }{E_{2}{}^{\prime }},\overset{\cdot }{E_{3}{}^{\prime }},\ldots \overset{\cdot }{,E_{m}{}^{\prime }}]$);

Step 2: Find the maximum first derivative of the first half U-shape for noise. Find the first value larger than the maximum and calculate the ratio of the first derivatives whose indexes are from 1 to the index of the first value to all first derivatives.

Step 3: Analyze the STE of the real-time data stream as step 1. The STE of the data stream is $E_{signal}=[E_{1},E_{2},E_{3},\ldots ,E_{n}]$. The STE is sorted in ascending order as $E_{signal}^{\prime }=[E_{1}{}^{\prime },E_{2}{}^{\prime },E_{3}{}^{\prime },\ldots ,E_{m}{}^{\prime }]$. The first derivatives are $\overset{\cdot }{E_{signal}^{\prime }}=[\overset{\cdot }{E_{1}{}^{\prime }},\overset{\cdot }{E_{2}{}^{\prime }},\overset{\cdot }{E_{3}{}^{\prime }},\ldots \overset{\cdot }{,E_{m}{}^{\prime }}]$. 

Step 4: Find the first value larger than the maximum first derivative of the first half U-shape for real-time data stream and calculate the quotient of index of the first value and the ratio obtained in step 2. The STE corresponding to the rounded quotient (the nearest integer) is determined as the threshold and the STE of the data stream larger than the threshold is judged as the signal. 

The core of the method is based on the statistical commonality of random noise. The distribution of the first derivatives of the noise’s ascending STE is U-shaped, and the statistical ratio (named Ratio1) of the first derivatives less than or equal to the maximum first derivative of the first half U-shape to all first derivatives is constant for any intensity noise. The first value larger than the maximum first derivative of the first half U-shape can be found. The statistical ratio (named Ratio2) of the first derivatives whose indexes are from 1 to the index of the first value to all first derivatives is constant for any intensity noise, where their proofs will be presented in Section 3. For the first derivatives of any real-time data stream’s ascending STE, the first value larger than the maximum first derivative of the first half U-shape belongs to the data stream’s noise because the STE of signal is larger than the STE of noise when SNR is larger than 0. Since the Ratio2 is constant for any intensity noise, the rounded quotient of the index of the first value and the constant Ratio2 is thus the top range of the total STE distribution of the data stream’s noise, which is the dividing point of the noise’s STE in the data stream’s ascending STE (the all U-shape distribution of the first derivatives of noise’s ascending STE is obtained for the first derivatives of real-time data’s ascending STE), and the STE corresponding to the index of the dividing point is the adaptive dividing level for signals and noise for every data stream at any SNR level shown in Figure 1.

## 3. Results

### 3.1. Results of Signal Activity Detection of the Simulated Database

This part explains the signal activity detection results of the simulated database in detail. The performance of the proposed method is compared with two representative detection methods: the LSFM [21] and the STFT methods [26]. The parameters of the three methods are shown in Table 1. A median filter is utilized to smooth the sequence obtained by the LSFM method, the STFT method and the proposed method and the smooth width is 10.

#### 3.1.1. Simulated Database Description

Three kinds of data streams with a total length of 150 min at five SNR levels (5 dB, 10 dB, 15 dB, 20 dB and 25 dB) compose the simulation database. In a data stream, every simulated vibration signal mentioned in Section 2 appears at one second and a kind of additive noise (white noise, pink noise and Brownian noise) with an SNR level is distributed between the interval of the signals. The total number of simulated signals with a time length of 0.1556 s and a maximum amplitude of 0.29 rad is 9000.

#### 3.1.2. Calculation of the Threshold

**Figure 2:**
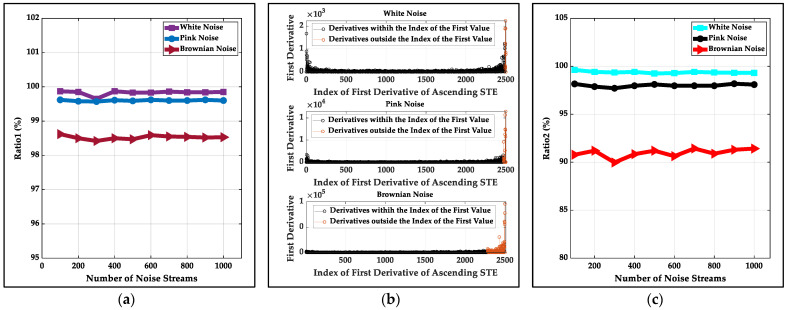
The characteristics of the first derivatives of three kinds of simulated noise’s ascending STE: (**a**) the statistical ratio (named Ratio1) of the first derivatives less than or equal to the maximum first derivative of the first half U-shape to all first derivatives; (**b**) the distribution of the first derivatives whose indexes are from 1 to the index of the first value; (**c**) the statistical ratio (named Ratio2) of the first derivatives whose indexes are from 1 to the index of the first value to all first derivatives.

The three methods have different threshold calculations. There is noise with the same SNR level at the beginning (time length is 10 s) for every simulated data stream at an SNR level. The thresholds of the LSFM and the STFT methods are calculated by the recommendations of the related literature with the known noise. For the proposed method, firstly, 1000 noise streams with a length of 10 s at 5 to 25 dB SNR are analyzed for a kind of noise, and the maximum first derivatives of the first half U-shape of three kinds of noise’s ascending STE are found and the Ratio1 mentioned in Section 2 is constant shown in Figure 2a (99.83%, 99.60% and 98.52% for white noise, pink noise and Brownian noise, respectively). The first value larger than the maximum first derivative also can be found and the first derivatives whose indexes are from 1 to the index of the first value are illustrated in Figure 2b, and the Ratio2 mentioned in Section 2 is constant (99.37%, 98.00% and 90.97% for white noise, pink noise and Brownian noise, respectively) shown in Figure 2c. Then, for every data stream with different noise, the threshold is updated by the first value larger than the maximum first derivative of the first half U-shape of the data stream’s ascending STE and is determined by the STE corresponding to the rounded quotient of the index of the first value and the different constant Ratio2.

#### 3.1.3. Performance Evaluation

A set of statistical measures are computed to qualify the quality of the three methods. At first, by totaling the number of detected signals versus the correct positions (referring to a total amount of true signals presenting in the data frame), classify the properly detected signals (true positive), missed signals (false negative) and mistake detected signals (false positive). Then, these indicators are obtained: accuracy (ACC, the ratio of true-positive detections against to all detected and not detected signals); false-discovery rate (FDR, the ratio of false-positive detections to the whole detected signals); false-negative rate (FNR, the ratio of false-negative detections to the sum of false-negative detections and true positive detections); and the F1-score (the harmonic of 1-FNR and 1-FDR) [34].

**Figure 3:**
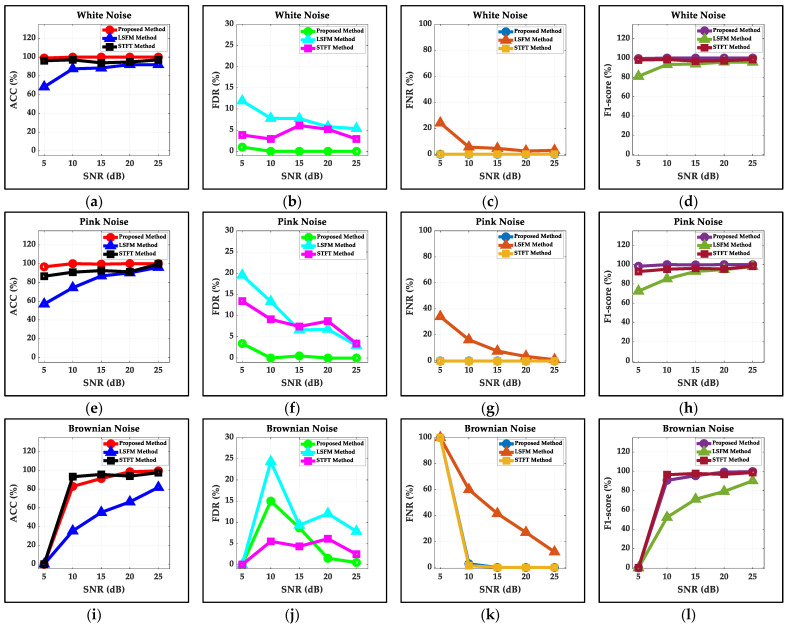
The statistical indicators of the LSFM method, the STFT method and the proposed method for three kinds of additive noise: (**a**–**d**) the statistical indicators of white noise; (**e**–**h**) the statistical indicators of pink noise; (**i**–**l**) the statistical indicators of Brownian noise.

As shown in Figure 3, the proposed method is compared with the LSFM and the STFT methods in terms of ACC, FDR, FNR and F1-score with three kinds of noise at 5 to 25 dB SNR. The proposed method outperforms the three methods (ACC:97.34%, FDR:1.25%, FNR:0.24% and F1-score: 98.6% on average for three kinds of noise at 5 to 25 dB SNR). The STFT method is superior to the other reference (ACC:94.17%, FDR:5.72%, FNR:0.12% and the F1-score: 96.97% on average for three kinds of noise at 5 to 25 dB SNR) due to the slight reduction in the flatness of the data stream’s Fourier spectrum when signals occur, calculated by the LSFM method. The proposed method based on the statistical commonality of random noise is superior to the better reference with known noise because the threshold of the proposed method updated by the first value larger than the maximum first derivative of the first half U-shape of real-time data stream’s ascending STE and determined by the STE corresponding to the rounded quotient of the index of the first value and the constant Ratio2 is more accurate. Three methods have unperfect statistical indicators at 5 dB SNR, especially when the signal is completely submerged by Brownian noise at 5 dB SNR, but the proposed method is still optimal for average statistical indicators.

**Figure 4:**
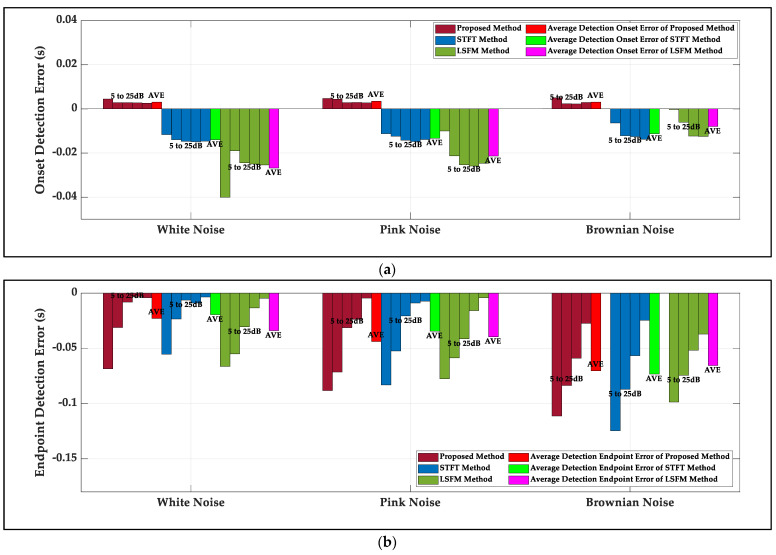
The onset and endpoint detection errors of the LSFM method, the STFT method and the proposed method at 5 to 25 dB SNR for three kinds of noise: (**a**) the onset detection errors of three methods at 5 to 25 dB SNR for three kinds of noise; (**b**) the endpoint detection errors of three methods at 5 to 25 dB SNR for three kinds of noise.

Figure 4 gives the onset detection errors (difference between detection onset and actual onset) and the endpoint detection errors (difference between detection endpoint and actual endpoint) at 5 to 25 dB SNR for three kinds of noise. The average length of signal is 0.1556 s. For the proposed method, the detected onset is behind the actual onset of 0.0032 s and the detected endpoint is ahead of the actual endpoint of 0.0457s on average for three kinds of noise. With the SNR level increasing, onset and endpoint detection errors of the proposed method go down. For the LSFM and the STFT methods, with the SNR level increasing, onset detection errors increase and endpoint detection errors decease for both methods based on long-window length leading to an earlier detected onset. Detected onsets are ahead of the actual onsets of 0.0187 s and 0.0129 s, while detected endpoints are ahead of the actual endpoints of 0.0463 s and 0.0427 s for the LSFM and the STFT methods on average for three kinds of noise, respectively. At a low SNR level, part of signal is submerged by noise, and the calculated endpoints of the proposed method (based on the time domain), the LSFM method (based on the frequency domain) and the STFT method (based on the time-frequency domain) are earlier. The proposed method has minimal onset detection error, and three methods have approximately equal endpoint detection errors.

### 3.2. Results of Signal Activity Detection of the Actual Database

#### 3.2.1. DAS System and Actual Database Description

**Figure 5:**
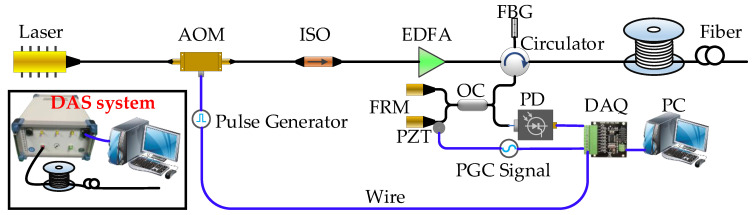
The structure of the DAS system based on the PGC algorithm (AOM: acousto-optic modulator; ISO: isolator; EDFA: erbium-doped optical fiber amplifier; FBG: fiber Bragg grating; OC: optical coupler; DAQ: data acquisition card; FRM: faraday rotation mirror; PZT: piezoelectric ceramic transducer; PD: photo detector; PC: personal computer).

The PGC algorithm-based DAS system is illustrated in detail in Figure 5 [35]. The coherent optical source is a narrow linewidth (3 KHz) laser with 20 mW continuous output at 1550.12 nm, which is pulse modulated by an AOM with the width of 30 ns and the repetition rate of 10 KHz. Then the pulsed lights are amplified by EDFA to get an appropriate peak power and an FBG is utilized to filter out the ASE noise. The pulse lights are propagating along the fiber and generate the coherent back Rayleigh scattered light carting sensing information which could interfere at the output of MI. The output of PD with 50 M of bandwidth is sampled by DAQ with a 200 MS/s sampling rate. The temporal sampling rate of the system is 5000 Hz and the frequency range is 50 to 2500 Hz, and its spatial resolution is 10 m.

**Figure 6:**
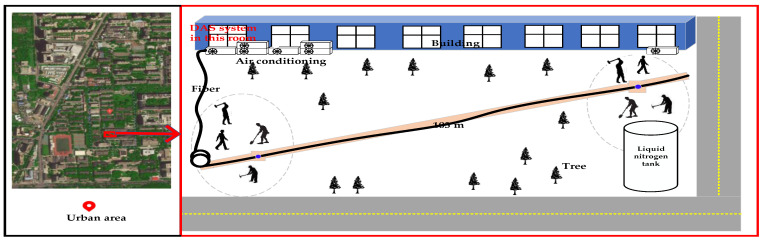
The field experiment in the Beijing urban environment.

In order to evaluate the proposed method, a field experiment in a Beijing urban environment with complex noise is arranged, shown in Figure 6. The DAS system is placed in the nearest laboratory, and the sensing fiber with a length of 103 m is buried 20–40 cm under the ground. The background noise in the left position (20 m from the starting of the buried fiber) is the superposition of air-conditioning noise and the complex urban noise and the background noise in the right position (20 m from the ending of the buried fiber) is the working noise of cryogenic liquid nitrogen tank superimposed with the complex urban noise. Four kinds of vibration signals with the bandwidth ranging from 50 to 2500 Hz are collected in every position as signal activity detection targets according to the time sequence which are digging the ground with a shovel, knocking the ground with a hammer, breaking up the ground with a pickaxe and walking of human. The signal acquisition time is from 7:00 p.m. to 10:20 p.m., the acquisition time of every signal is 25 min and the total number of four kinds of signals is 1920, 3254, 1838 and 3340, in that order.

#### 3.2.2. Calculation of Threshold

**Figure 7:**
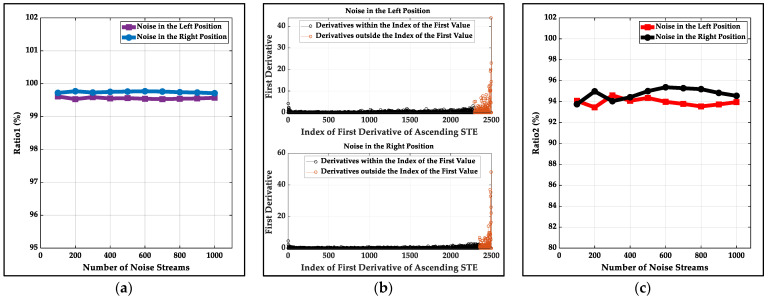
The characteristic of the first derivatives of environmental noise’s ascending STE: (**a**) the statistical ratio (named Ratio1) of the first derivatives less than or equal to the maximum first derivative of the first half U-shape to all first derivatives; (**b**) the distribution of first derivatives whose indexes are from 1 to the index of the first value; (**c**) the statistical ratio (named Ratio2) of the first derivatives whose indexes are from 1 to the index of the first value to all first derivatives.

For the actual database, 1000 noise streams with a length of 10 s are collected in the left and right positions. The thresholds of the LSFM and the STFT methods are initialized and updated by the recommendations of the related literature with the noise. For the proposed method, firstly, the noise streams are analyzed for every position, and the maximum first derivative of the first half U-shape of noise’s ascending STE is found and the statistical ratio (named Ratio1) of the first derivatives less than or equal to the maximum first derivative to all first derivatives is constant shown in Figure 7a (99.56% and 99.73% for the noise of two positions). The first value larger than the maximum first derivative also can be found, and the first derivatives whose indexes are from 1 to the index of the first value are illustrated in Figure 7b. The statistical ratio (named Ratio2) of the first derivatives whose indexes are from 1 to the index of the first value to all first derivatives is constant (94.00% and 94.68% for noise of two positions) and is shown in Figure 7c. Then, the threshold is updated by the first value larger than the maximum first derivative of the first half U-shape of the data stream’s ascending STE and is determined by the STE corresponding to the rounded quotient of the index of the first value and the constant ratio (94.00% and 94.68%) for every position data stream.

#### 3.2.3. Performance Evaluation

**Figure 8:**
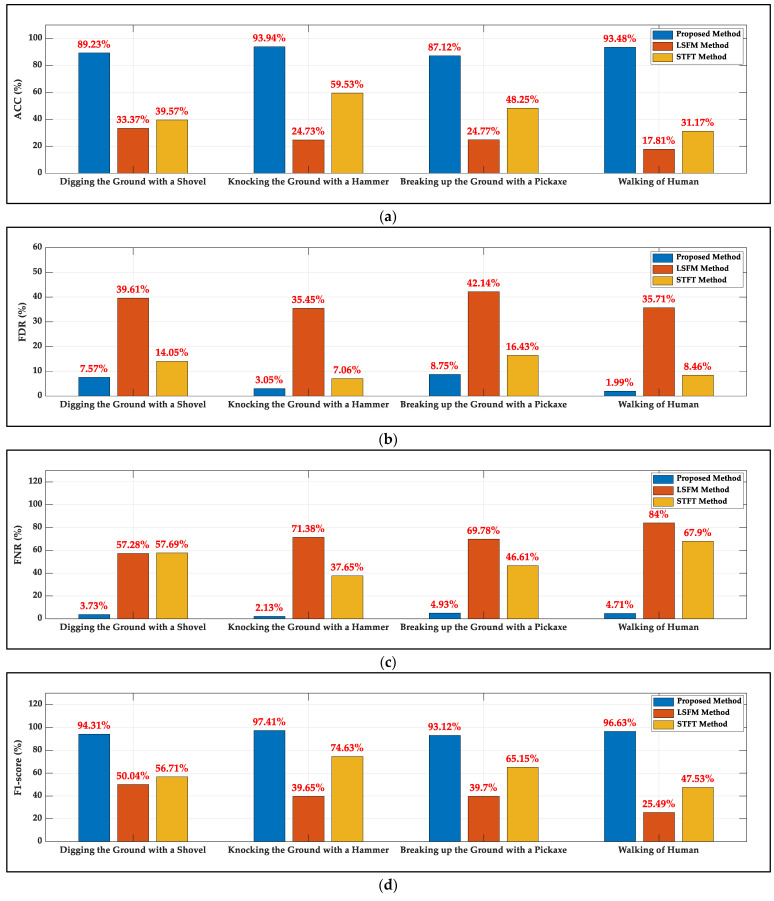
The statistical indicators of the proposed method, the LSFM method and the STFT method for actual database: (**a**) ACC; (**b**) FDR; (**c**) FNR; (**d**) F1-score.

Figure 8 shows the statistical indicators of three methods for the four signals. It is clear that the proposed method provides the best performance among the three methods for the complex urban noise (ACC: 90.94%, FDR: 5.34%, FNR: 3.88% and F1-score: 95.37% on average of four signals). For the LSFM method, a slight reduction in the flatness of the data stream’s Fourier spectrum is calculated when signals occur due to the urban noise changes significantly within 10 s and the FDR and the FNR indicators are high. The STFT method is better than the LSFM method. For the actual data, the initial threshold chosen by the STFT method is based on the varying environmental noise at the beginning of data stream and the threshold is updated by statistics of signal amplitude, which leads to poor statistical indicators. The proposed method is compared with the better reference for the four signals. It can be observed that on average, the proposed method is superior to the better reference in terms of the ACC (46.31%), FDR (6.16%), FNR (48.59%) and F1-score (34.36%) due to the accurate adaptability of the threshold calculated by statistical commonality of the complex urban noise, and the bottleneck problem for current methods of calculating the threshold separating signals and environmental noise accurately without affecting the integrity of signal is solved.

**Figure 9:**
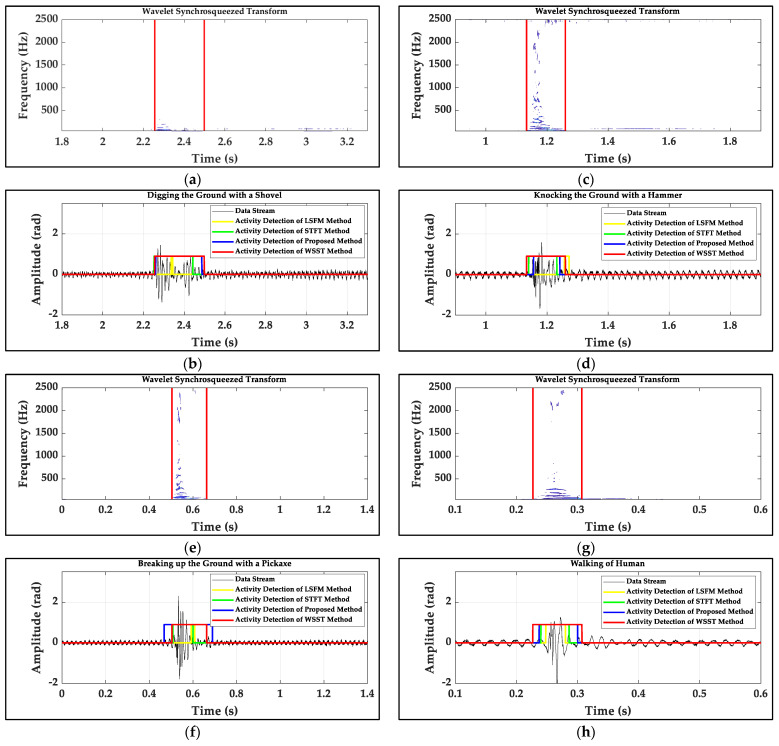
The manual determination of the onsets and the endpoints of four kinds of actual signals with the wavelet synchrosqueezed transform method and the comparation of detection onset and endpoint errors obtained by proposed method, the LSFM method and the STFT method: (**a**,**b**) digging the ground with a shovel; (**c**,**d**) knocking the ground with a hammer; (**e**,**f**) breaking up the ground with a pickaxe; (**g**,**h**) walking of human.

**Figure 10:**
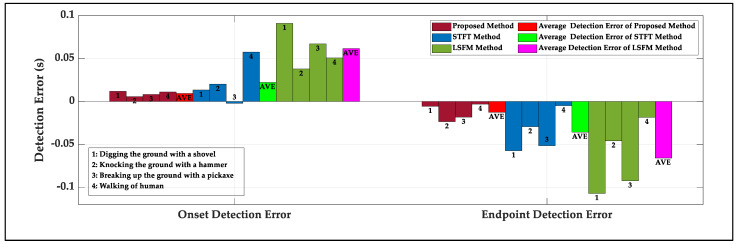
The detection onset and endpoint errors of the four kinds of signals for the proposed method, the LSFM method and the STFT method.

Figure 9 gives an example of the manual determination of the onset and the endpoint of the actual signal with the wavelet synchrosqueezed transform (WSST) method, which are chosen as the actual onset and the actual endpoint of actual signal [34], and the error calculations of the onset and endpoint are the same as the simulated data stream. Due to the limitation of the number of correct detections by the LSFM method, 100 samples are collected to calculate the errors of the onset and endpoint for a kind of signal shown in Figure 10. The average time length of the four signals is 0.2087 s. For the LSFM and the STFT methods, for high accuracy, the initial thresholds have to choose the maximum of the beginning noise. Two methods yield more significant errors, especially for the LSFM method which has the maximum error. For the STFT method, the detected onset is behind of the actual onset of 0.0223 s and the detected endpoint is ahead of the actual endpoint of 0.0357 s on average for four kinds of signals. Compared to the better reference, the onset and endpoint detected by the proposed method are 0.013 s and 0.0231 s closer to the actual points, respectively, which demonstrates the superiority of the method.

**Figure 11:**
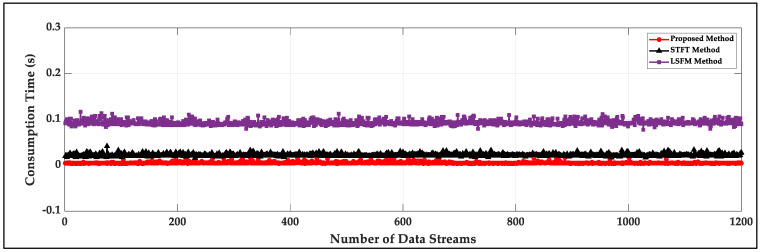
The consumption time of signal activity detection for the proposed method, the LSFM method and the STFT method.

Three approaches are implemented through the software scripts executed by MATLAB^®^ R2020a in a PC with a CPU Intel Core i9-10980XE @ 3.0 GHz(manufacturer of Intel, made in Portland, OR, USA) and 32 GB RAM. A total of 1200 data streams with a time length of 10 s are calculated and the average consumption time of the proposed method (including threshold calculation and signal activity detection) for every data stream is 0.0057 s, shown in Figure 11. The average consumption times of the STFT method (computation complexity generated by short-time Fourier transform) and the LSFM method (computation complexity generated by dividing the geometric mean of the power spectrum by the arithmetic mean of the power spectrum) are 0.0224 s and 0.09343 s, respectively, which can prove that the proposed method is very highly efficient.

## 4. Discussion

**Figure 12:**
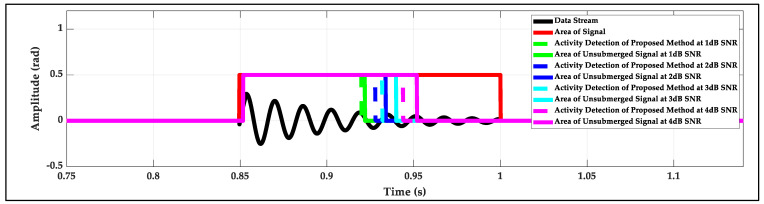
The detected endpoint errors of the proposed method at 1 to 4 dB SNR.

At a low SNR level, part of the signal is inevitably submerged by noise, and the detection endpoints of the proposed method (based on time domain), the LSFM method (based on frequency domain) and the STFT method (based on time-frequency domain) have approximately equal errors, which are earlier than the actual endpoint. Figure 12 shows the detection endpoint error of the proposed method for a simulated signal with the maximum amplitude of 0.29 rad and the duration of 0.1556 s at 1 to 4 dB SNR in detail. At 1 dB, 2 dB, 3 dB and 4 dB SNR, the durations of unsubmerged signal are 0.0722 s, 0.0843 s, 0.0902 s and 0.1022 s, respectively, and the detected endpoints are ahead of actual endpoint of the unsubmerged signal of 0.002 s, 0.002 s, 0.008 s and 0.008 s, respectively. For all detection methods, if the features of the signal are submerged by noise, the signal will not be effectively detected [19]. The method of signal feature extraction which can characterize the signal more prominent at low SNR may be a way to solve the problem that is my next research work.

## 5. Conclusions

In this work, a novel signal activity detection method is proposed with the adaptive-calculated threshold based on the statistical commonality of random noise, which is the distribution of the first derivatives of noise’s short-term energy (STE) in ascending order is U-shaped and the statistical ratio of the first derivatives whose indexes are from 1 to the index of the first value (the first value larger than the maximum first derivative of the first half of the U-shape of random noise’s ascending STE is named the first value) is constant for any intensity of noise. The threshold is the adaptive dividing level of signals and noise that is updated by the first value and the constant ratio. Experiments with simulated and urban field databases are implemented. For the simulated database, the proposed method has optimal statistical indicators of signal activity detection (ACC: 97.34%, FDR: 1.25%, FNR: 0.24%, and F1-score: 98.6% on average for white noise, pink noise and Brownian noise at 5 to 25 dB SNR). For the simulated signal with a time length of 0.1556 s and a maximum amplitude of 0.29 rad, the proposed method has the minimum average detected onset error (0.0032 s) and the average detected endpoint error is approximately equal to the reference methods for three kinds of noise at 5 to 25 dB SNR. For field database with complex urban noise, the proposed method yields the good statistical indicators of signal activity detection (ACC: 90.94%, FDR: 5.34%, FNR: 3.88% and F1-score: 95.37% on average of the four kinds of signals), while the STFT and the LSFM methods have poor statistical indicators. For the four kinds of signals with the average time length of 0.2087 s, the proposed method also has the minimum average detected onset error (0.0093 s) and the minimum average detected endpoint error (0.0126 s). The consumption time of the proposed method is only 0.0057 s for a data stream with a length of 10 s. It is proven that the proposed method is accurate, efficient for signal activity detection of the data stream measured by DAS, which can be applied to signal-detection-related applications.

Our future work will focus on how to realize the signal activity detection of the data stream measured by DAS at low SNR level accurately and efficiently.

## Figures and Tables

**Table 1:** The parameters of the three kinds of signal activity detection methods.

Method	Window Function	Data Stream Length (s)	Window Length (ms)	Overlap Length (ms)	Smooth Width
LSFM	Rectangular window	10	40	36	10
STFT	Rectangular window	10	20	16	10
Proposed method	Rectangular window	10	4	0	10

## Appendix A

This part mainly explains the rationality of the simulation data applied to signal activity detection. Four kinds of ground vibrations collected in this article are simulated (digging the ground with a shovel, knocking the ground with a hammer, breaking up the ground with a pickaxe and walking of a human). Every vibration is divided into two parts with the maximum displacement as the dividing point shown in Figure A1. The first part is the displacement caused by incident loading and the second part is underdamped motion during unloading. The parameters of the first part are calculated by fitting the actual displacement with the polynomial of loading state in (1) with 95% R-Squared, and the parameters of the second part are computed by the polynomial of unloading state in (1) with the method of measuring the frequency and damping coefficient for an engineering system [36]. By comparing with the actual displacements of the four kinds of actual vibrations, although there is a bit mismatch between the calculated motion and the actual vibrations in the middle, it is suitable for signal activity detection.
